# Modified Polyadenylation-Based RT-qPCR Increases Selectivity of Amplification of 3′-MicroRNA Isoforms

**DOI:** 10.3389/fgene.2018.00011

**Published:** 2018-01-24

**Authors:** Charlotte Nejad, Geneviève Pépin, Mark A. Behlke, Michael P. Gantier

**Affiliations:** ^1^Centre for Innate Immunity and Infectious Diseases, Hudson Institute of Medical Research, Clayton, VIC, Australia; ^2^Department of Molecular and Translational Science, Monash University, Clayton, VIC, Australia; ^3^Integrated DNA Technologies Inc., Coralville, IA, United States

**Keywords:** microRNA isoforms, isomiR, polyadenylation, RT-qPCR, selective amplification

## Abstract

MicroRNA (miRNA) detection by reverse transcription (RT) quantitative real-time PCR (RT-qPCR) is the most popular method currently used to measure miRNA expression. Although the majority of miRNA families are constituted of several 3′-end length variants (“isomiRs”), little attention has been paid to their differential detection by RT-qPCR. However, recent evidence indicates that 3′-end miRNA isoforms can exhibit 3′-length specific regulatory functions, underlining the need to develop strategies to differentiate 3′-isomiRs by RT-qPCR approaches. We demonstrate here that polyadenylation-based RT-qPCR strategies targeted to 20–21 nt isoforms amplify entire miRNA families, but that primers targeted to >22 nt isoforms were specific to >21 nt isoforms. Based on this observation, we developed a simple method to increase selectivity of polyadenylation-based RT-qPCR assays toward shorter isoforms, and demonstrate its capacity to help distinguish short RNAs from longer ones, using synthetic RNAs and biological samples with altered isomiR stoichiometry. Our approach can be adapted to many polyadenylation-based RT-qPCR technologies already exiting, providing a convenient way to distinguish long and short 3′-isomiRs.

## Introduction

MicroRNAs (miRNAs) are short RNAs controlling the translation of target messenger RNAs (mRNAs). They are processed from hairpin-like transcripts to their mature form through a sequential cleavage operated by Drosha in the nucleus, and Dicer, in the cytoplasm (Ha and Kim, 2014). Mature miRNA intracellular levels are under stringent control, as inefficient miRNA biogenesis and the resulting global decrease of miRNA levels are directly associated with the development of tumor cells (Melo et al., 2010; Wu et al., 2013). Conversely, however, accumulation of select miRNAs can also promote cancer development through the coordinated action on tumor suppressors such as Pten, or pro-inflammatory pathways such as NF-κB (Garofalo et al., 2009; Olive et al., 2009; Galardi et al., 2011; Gantier et al., 2012; Liu et al., 2014). Intracellular miRNA levels are therefore tightly controlled through the modulation of their expression and processing, with as many as 180 binding proteins interacting with select precursor miRNAs (pre-miRNAs) recently identified (Treiber et al., 2017).

While pre-miRNA-binding proteins can control the processing of Dicer and Drosha, they also have the capacity to influence how the pre-miRNAs are cleaved, directly impacting on the 5′-end and 3′-end length of the mature miRNA (Lee et al., 2013). Such processing variations resulting in miRNA isoforms (referred to as templated isomiRs) are very frequently observed (Neilsen et al., 2012; Tan et al., 2014; Siddle et al., 2015; Karali et al., 2016; Juzenas et al., 2017; Telonis et al., 2017), and significantly broaden the landscape of miRNA molecules existing in a cell. This may help identify disease-specific isoforms, which could potentially be developed as novel biomarkers (Telonis et al., 2017). Critically, both 5′ and 3′-length variations have been linked to different biological functions, emphasizing their functional importance (Tan et al., 2014; Yu et al., 2017).

miRNAs can be detected through many different technologies, including small RNA-sequencing (RNA-Seq), microarrays, RT-qPCR approaches, nCounter^®^ Nanostring, and northern blot, among others. Each miRNA detection technique has its strengths and weaknesses, but RT-qPCR has been found to be the most sensitive approach to quantify circulating miRNAs (Mestdagh et al., 2014), favoring its use in biomarkers studies. RT-qPCR remains the most popular technique used to date for its ease of use and low-cost. Importantly, however, the capacity of RT-qPCR approaches to distinguish between 3′-isomiRs remains poorly defined, with prior reports indicating that RT-qPCR approaches only poorly distinguish 3′-isomiRs with ±1 base variation (Wu et al., 2007; Schamberger and Orban, 2014; Magee et al., 2017).

The present work describes a simple approach amenable to widely used polyadenylation-based RT-qPCR protocols, conferring increased selectivity toward shorter isoforms. We demonstrate its usefulness on synthetic RNAs and biological samples with naturally altered isomiR stoichiometry.

## Materials and Methods

### Cell Culture

Human hTERT BJ fibroblasts (referred to as human fibroblasts herein – gift from V. Hornung, Ludwig-Maximilians-University), were grown in DMEM (Life Technologies) supplemented with 10% sterile fetal bovine serum (Life Technologies), 1 mM sodium pyruvate and 1× antibiotic/antimycotic (Life Technologies) (referred to as complete DMEM). 80,000 human fibroblasts were plated in a 24-well plate, and stimulated with human IFN-β for 24 h. Bone marrow derived macrophages (BMDMs) from C57BL/6 wild-type mice were generated as previously described (Ferrand and Gantier, 2016), and stimulated in 20% L-929 condition medium on day 7 of differentiation with lipopolysaccharide (LPS) from *Escherichia coli* Serotype O111:B4 (TLR4 agonist, Enzo Life Sciences) or recombinant mouse IFN-β (Stifter et al., 2014) (gift from N. A. de Weerd and P. J. Hertzog, Hudson Institute). Recombinant human IFN-β (Rebif, Merck Serono) was used at a final activity of 1000 IU/ml.

### Reverse Transcription Quantitative Real-Time PCR (RT-qPCR)

Total RNA from human fibroblasts or BMDMs was purified using the GenElute Total RNA Purification kit (Sigma). Synthetic miRNAs and RNAs were synthesized as single-stranded RNAs by Integrated DNA Technologies (IDT), and resuspended in duplex buffer (100 mM potassium acetate, 30 mM HEPES, pH 7.5, DNase–RNase free H_2_O)—these were directly polyadenylated and reverse transcribed, without being transfected into cells. For polyadenylation detection, the Mir-X miRNA First-Strand Synthesis kit (Clontech) was used on total RNA or synthetic miRNA/RNA according to the manufacturer’s instructions. Briefly, 1–8 μg of total RNA or 2.25 pmol of synthetic miRNA/RNA in a total volume of 3.75 μL was combined with 5 μL of mRQ Buffer and 1.25 μL of mRQ Enzyme. The mixture was incubated for 1 h at 37°C and the reaction was stopped after incubation at 85°C for 5 min. Fifteen microliter of RNase and DNase free water was added to the reverse-transcribed polyadenylated total cellular RNA, and 1 μL of the resulting mix was used per qPCR reaction with the Power SYBR Green mastermix (Applied Biosystems). The reverse-transcribed polyadenylated synthetic miRNA/RNA reaction was diluted 1/100 in RNase and DNase free water, prior to qPCR analysis. The mRQ 3′ Primer (Clontech) was used as reverse primer in all Mir-X cDNA qPCRs. The U6 RNA forward primer used for Mir-X cDNA qPCRs was provided in the Mir-X kit and was used as reference small RNAs using the 2-^ΔΔCq^ method. For detection with the miRCURY LNA hsa-miR-222-3p (YP00204551 – targeted to the 21 nt isoform), 1.125 pmol of synthetic miRNA was polyadenylated and reverse transcribed with the miRCURY LNA RT Kit, following the manufacturer’s instructions (Qiagen). The reverse-transcribed polyadenylated synthetic miRNA reaction was diluted 1/100 in RNase and DNase free water, prior to qPCR analysis. One microliter of the resulting dilution was used per qPCR reaction with the Power SYBR Green mastermix (Applied Biosystems). Stem-loop hsa-miR222-3p TaqMan^®^ assays (Applied Biosystems) were used according to the manufacturer’s instructions, where 3.2 fmol of synthetic miRNA was reverse transcribed with specific reverse transcription primers. The manufacturer’s references of the assays used are: miR-222-3p (#2276 and #525 – targeted to the 21 nt and the 24 nt isoforms, respectively). One microliter of the resulting cDNA was amplified with the SensiFAST Probe Hi-ROX Kit (Bioline). All RT-qPCRs were carried out on the HT7900 RT-PCR system (Applied Biosystems). Relative amplification of synthetic miRNAs/RNA was calculated using 2-^ΔCq^, relative to the indicated Cq. Each RT-qPCR was carried out in technical duplicate. Melting curves were used in each run to confirm specificity of amplification. The synthetic RNAs are listed in **Table 1**, while the primers used are listed in **Table 2**.

**Table 1:** Synthetic RNAs used in the study.

RNA name	Sequence (5′-3′)
miR-221-20 nt	rArGrCrUrArCrArUrUrGrUrCrUrGrCrUrGrGrGrU
miR-221-23 nt	rArGrCrUrArCrArUrUrGrUrCrUrGrCrUrGrGrGrUrUrUrC
miR-222-21 nt	rArGrCrUrArCrArUrCrUrGrGrCrUrArCrUrGrGrGrU
miR-222-22 nt	rArGrCrUrArCrArUrCrUrGrGrCrUrArCrUrGrGrGrUrC
miR-222-23 nt	rArGrCrUrArCrArUrCrUrGrGrCrUrArCrUrGrGrGrUrCrU
miR-222-24 nt	rArGrCrUrArCrArUrCrUrGrGrCrUrArCrUrGrGrGrUrCrUrC
miR-222-25 nt	rArGrCrUrArCrArUrCrUrGrGrCrUrArCrUrGrGrGrUrCrUrCrU
RNA#1	rGrArArGrGrArGrGrGrUrGrArCrCrUrGrArUrArArArCrCrArA
RNA#2	rArCrUrCrCrUrUrCrArUrUrCrUrCrCrCrUrUrUrCrArArArGrGrCrU
RNA#3	rGrArGrGrUrUrUrArGrGrUrArUrCrGrArArGrUrUrGrGrGrUrCrArA
RNA#4	rCrArGrArArCrArArArGrGrCrArUrCrGrUrUrGrGrArGrUrUrCrArG
RNA#5	rArGrUrArUrCrUrCrArArCrArGrCrUrArArUrUrUrGrGrCrUrGrCrG
RNA#6	rGrArArGrGrArGrGrGrUrGrArCrCrUrGrArUrArGrGrUrUrArC
RNA#7	rArGrCrArGrCrUrArUrCrArGrGrUrCrArCrCrCrUrCrCrUrUrCrUrU
RNA#7-MIS	rArGrCrArGrCrUrArUrCrArGrGrUrArArArCrCrUrCrCrUrUrCrUrU

*rX denotes RNA bases.*

**Table 2:** DNA primers used in the study.

Forward primer name	Sequence (5′-3′)
F222-25	AGCTACATCTGGCTACTGGGTCTCT
F222-24	AGCTACATCTGGCTACTGGGTCTC
F222-23	AGCTACATCTGGCTACTGGGTCT
F222-22	AGCTACATCTGGCTACTGGGTC
F222-21	AGCTACATCTGGCTACTGGGT
F221-23	AGCTACATTGTCTGCTGGGTTTC
F221-20	AGCTACATTGTCTGCTGGGT
F221-20-4A	AGCTACATTGTCTGCTGGGTAAAA
F222-21-2A	AGCTACATCTGGCTACTGGGTAA
F222-21-5A	AGCTACATCTGGCTACTGGGTAAAAA
F1-25	GAAGGAGGGTGACCTGATAAACCAA
F1-21	GAAGGAGGGTGACCTGATAAA
F1-21-4A	GAAGGAGGGTGACCTGATAAAAAAA
F222-25-4A	AGCTACATCTGGCTACTGGGTCTCTAAAA
F222-24-4A	AGCTACATCTGGCTACTGGGTCTCAAAA
F222-23-4A	AGCTACATCTGGCTACTGGGTCTAAAA
F222-22-4A	AGCTACATCTGGCTACTGGGTCAAAA
F222-21-4A	AGCTACATCTGGCTACTGGGTAAAA
F199a-20	CCCAGTGTTCAGACTACCTG
F199a-20-4A	CCCAGTGTTCAGACTACCTGAAAA
F199a-23	CCCAGTGTTCAGACTACCTGTTC
F2-27	ACTCCTTCATTCTCCCTTTCAAAGGCT
F2-22	ACTCCTTCATTCTCCCTTTCAA
F2-22-4A	ACTCCTTCATTCTCCCTTTCAAAAAA
F3-27	GAGGTTTAGGTATCGAAGTTGGGTCAA
F3-22	GAGGTTTAGGTATCGAAGTTGG
F3-22-4A	GAGGTTTAGGTATCGAAGTTGGAAAA
F4-27	CAGAACAAAGGCATCGTTGGAGTTCAG
F4-22	CAGAACAAAGGCATCGTTGGAG
F4-22-4A	CAGAACAAAGGCATCGTTGGAGAAAA
F5-27	AGTATCTCAACAGCTAATTTGGCTGCG
F5-22	AGTATCTCAACAGCTAATTTGG
F5-22-4A	AGTATCTCAACAGCTAATTTGGAAAA
F6-25	GAAGGAGGGTGACCTGATAGGTTAC
F6-21	GAAGGAGGGTGACCTGATAGG
F6-21-4A	GAAGGAGGGTGACCTGATAGGAAAA
F7-27	AGCAGCTATCAGGTCACCCTCCTTCTT
F7-22	AGCAGCTATCAGGTCACCCTCC
F7-22-4A	AGCAGCTATCAGGTCACCCTCCAAAA
F7MIS-27	AGCAGCTATCAGGTAAACCTCCTTCTT
F7MIS-22	AGCAGCTATCAGGTAAACCTCC
F7MIS-22-4A	AGCAGCTATCAGGTAAACCTCCAAAA
miR-221-3p MySEQ	CCTACACGACGCTCTTCCG ATCTAGCTACATTGTCTGCTGGG
snoRNA-202 MySEQ	CCTACACGACGCTCTTCCGATCTGC TGTACTGACTTGATGAA AGTAC

### Small RNA-Seq Library Preparation and RNA Sequencing

Small RNA libraries from human fibroblasts treated or not with IFN-β for 24 h were made using the NEBNext Small RNA Library Prep Set for Illumina (New England Biolabs) according to the manufacturer’s instructions and library quality was analyzed using an Agilent Bioanalyzer 2100 (Agilent Technologies) (Nejad et al., 2018). The libraries were sequenced on a NextSeq 500 machine at the ACRF Cancer Genomics Facility to produce single end, 50 base pair reads. Adapter-trimmed FASTQ files have been deposited in the EBI European Nucleotide Archive (PRJEB22632). Targeted amplification of miR-221-3p and snoRNA 202 was carried out using total RNA from BMDM using modified PAT-seq (Harrison et al., 2015), with miR-221-3p MySEQ and snoRNA-202 MySEQ forward primers. miRNA isoforms were identified using an in house perl script (Nejad et al., 2018). For each microRNA, read alignments which overlapped the mature microRNA’s genomic locus were classified and counted according to the start and end positions of the alignments.

### Statistical Analyses

Statistical analyses were carried out using Prism 7 (GraphPad Software Inc.). Two-tailed unpaired *t*-tests and non-parametric Mann–Whitney *U*-tests were used to compare pairs of conditions, when appropriate. Symbols used: ^∗^*P* ≤ 0.05, ^∗∗^*P* ≤ 0.01, ^∗∗∗^*P* ≤ 0.001, ^∗∗∗∗^*P* ≤ 0.0001. ns, not significant.

## Results

### Conventional Polyadenylation RT-qPCR Exhibits Specificity toward Long IsomiRs

**FIGURE 1:**
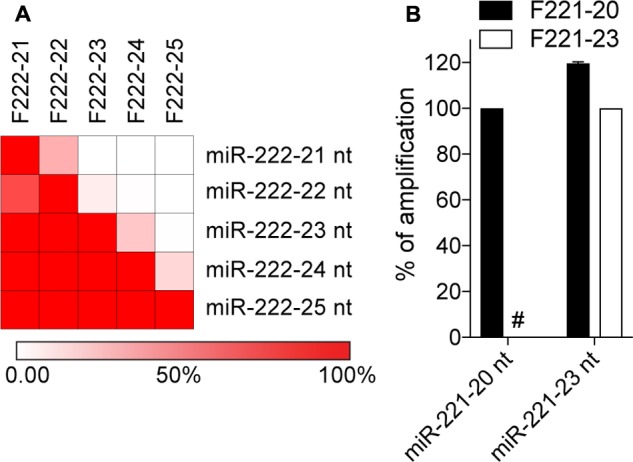
Polyadenylation RT-qPCR exhibits specificity toward long isomiRs. Synthetic miRNA variants of miR-222-3p **(A)** and miR-221-3p **(B)** were analyzed by polyadenylation RT-qPCR. Synthetic miRNAs 3′-end length variants are referred to by their name, with their length appended. The forward primer names start with an “F”, and are ended by the length of the isomiR there are designed to amplify. The amplification values on the heatmap **(A)** are normalized per line, to the amplification obtained with the primer targeting the isomiR on this line. Red = amplified; White = not amplified. The reddest color reflects values of 100% or more. **(B)** The amplification values are normalized per isomiR, to the Cq value obtained for the primer targeting the isomiR, and are displayed as percentages (mean ± SEM is shown). **(A,B)** Data shown is averaged from two independent RT-qPCR analyses. #: denotes amplification values smaller that 0.1%.

It has previously been suggested that qPCR approaches relying on stem-loop or polyadenylation reverse transcription do not have the capacity to distinguish miRNA isoforms differing in their 3′-end (Wu et al., 2007; Schamberger and Orban, 2014; Magee et al., 2017). However, we hypothesized that primers targeted to longer miRNA isoforms should have limited capacity to amplify shorter isomiRs, due to a lack of binding of the primer 3′-end, which is essential in 5′-3′ polymerase amplification (Simsek and Adnan, 2000). Given that templated miRNA isoforms can vary greatly in length, we decided to test the capacity of polyadenylated reverse transcribed synthetic miR-222-3p variants ranging from 21 to 25 nt (Yu et al., 2017), to be detected by a range of forward primers directly matching the isoform targeted (**Figure 1A**). Not too surprisingly, forward primers targeted to the shorter isoforms could all amplify the longer ones, which were anticipated since the longer isomiRs have perfect binding sites for these primers. Nonetheless, forward primers targeted to longer isomiRs showed clear selectivity toward these isoforms, when compared to shorter ones (**Figure 1A**). As such, forward primers could not amplify isomiRs lacking 2 or more nucleotides at the 3′-end (**Figure 1A**). A similar observation was made with synthetic miR-221-3p 20 and 23 nt isomiRs (**Figure 1B**), confirming that the lack of perfect annealing of the forward primer 3′-end to shorter isoforms strongly impacted their amplification, and that selectivity could be achieved toward the amplification of longer 3′-isomiRs over shorter ones.

### Forward Primer 3′-Extension Increases Selectivity toward Short IsomiRs

**FIGURE 2:**
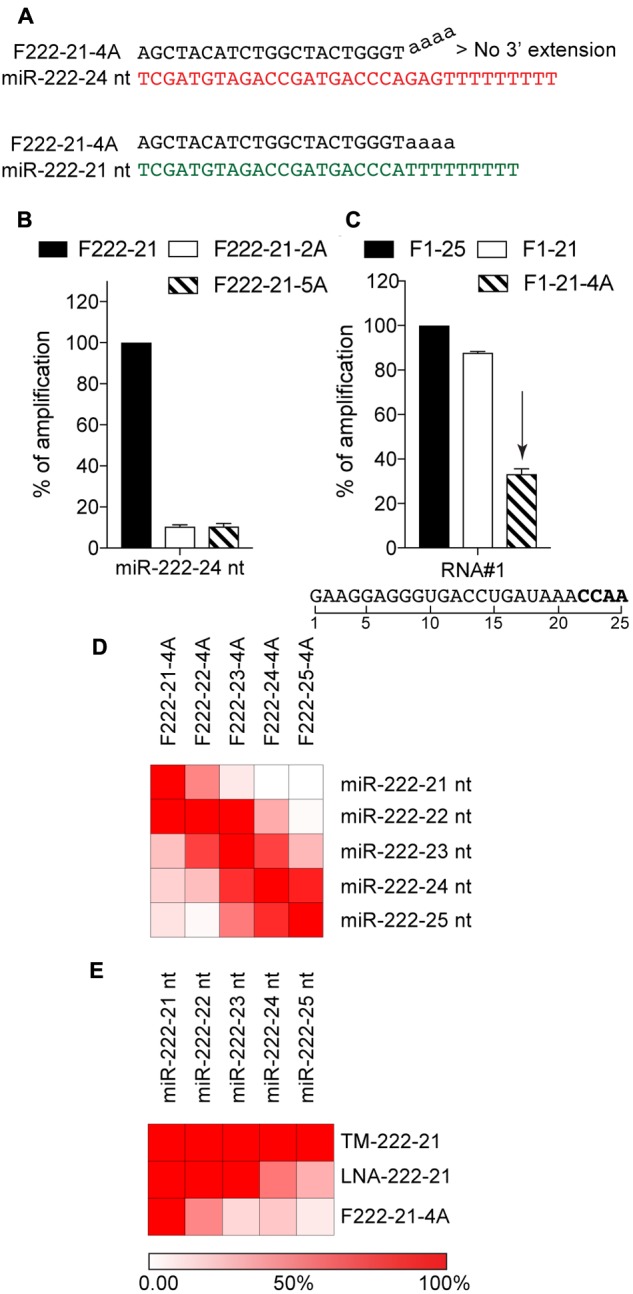
Forward primer 3′-extension increases selectivity toward short isomiRs. **(A)** Schematic of the selective amplification of poly-A reversed-transcribed 21 nt (in green) or 24 nt (in red) miR-222-3p isomiRs using a PCR approach with a forward primer of 21 nt comprising a stretch of 4 adenosines in its 3′-end. The 4A stretch creates a structural distortion in the 3′-end of the primer that is not favorable to 5′-3′ extension by Taq polymerase when the primer binds to the 24 nt miRNA variant. **(B)** Polyadenylated, reverse-transcribed miR-221-3p 24 nt isomiR was amplified with a non-modified 21 nt forward primer (F222-21), or two 21 nt forward primers with 2 or 5 terminal adenosines in their 3′-end (F222-21-2A and F222-21-5A). The amplification values are normalized to the Cq value obtained for the non-modified 21 nt primer, and are displayed as percentages (mean ± SEM is shown). **(C)** Amplification of the polyadenylated, reverse transcribed synthetic 25 nt RNA#1, which contains a 3′-end with a high proportion of adenosine residues. The amplification values are normalized to the Cq value obtained for the non-modified 25 nt primer, and are displayed as percentages (mean ± SEM is shown). **(D)** Synthetic miRNA variants of miR-222-3p were analyzed by polyadenylation RT-qPCR with 4A-modified forward primers of different length. **(E)** Synthetic miRNA variants of miR-222-3p were analyzed by polyadenylation RT-qPCR with 4A-modified forward (F222-21-4A), Taqman stem loop assay (TM-222-21) or miRCURY LNA assay (LNA-222-21), all targeted to the 21 nt isoform. **(D,E)** The amplification values on the heatmap are normalized per line, to the amplification obtained with the primer targeting the isomiR on this line **(D)** or the 21 nt isoform **(E)**. Red = amplified; White = not amplified. The reddest color reflects values of 100% or more. **(C–E)** The forward primer names start with an “F”, and are ended by the length of the isomiR there are designed to amplify, with “4A” denoting a 4A 3′-terminal stretch. **(B–E)** Data shown is averaged from two independent RT-qPCR analyses.

The previous observation led us to speculate that alteration of the forward primer 3′-end, may be used to confer increased selectivity toward shorter isomiRs (**Figure 2A**). We decided to make use of the poly-A sequence which is added during the 3′-end polyadenylation, and tested the impact of 2 and 5 “A” added to the 3′-end of the primer targeted to miR-222-3p 21 nt, on the amplification of the miR-222-3p 24 nt isoform—reasoning that the 3′-structural distortion created when binding to longer isoforms would dampen their amplification (**Figure 2A**). This approach confirmed that modification of the 3′-end could be used to limit amplification of the longer isoforms by >80%, with comparable results independent of the amount of A residues added (**Figure 2B**), possibly pertaining to the importance of the last few 3′-end residues in 5′-3′ polymerase activity (Simsek and Adnan, 2000). We opted for an addition of 4 “A” in further experiments (referred to as 4A-modification hereafter), under the assumption that it would allow better discrimination of longer RNAs with A-rich 3′-end. In line with this, amplification of a polyadenylated reverse transcribed 25 nt RNA#1 containing an “AAACCAA” 3′-end was decreased by more than 60% with the 4A-modification (**Figure 2C**). To confirm the performance of the 4A-modification, we next assessed its selectivity on our panel of 21–25 nt miR-222-3p variants (**Figure 2D**). Modified forward primers showed a decreased capacity to amplify isomiRs with >2 nt additional bases at the 3′-end (**Figure 2D**), supporting that increased specificity toward shorter isomiRs could be achieved with the 4A-modification of polyadenylation RT-qPCR (compare **Figures 1A**, **2D**). In addition, we compared the amplification of our panel of 21–25 nt miR-222-3p variants by our 4A-modified miR-222-3p 21 nt primer approach, to that by miRCURY LNA and stem-loop miRNA TaqMan^®^ assays, targeted to the 21 nt miR-222-3p isoform (**Figure 2E**). This analysis revealed that the 4A-modified approach was the most specific toward the 21 nt isoform (**Figure 2E**).

### 4A-Modification Decreases Off-Target Amplification of Long-IsomiRs

**FIGURE 3:**
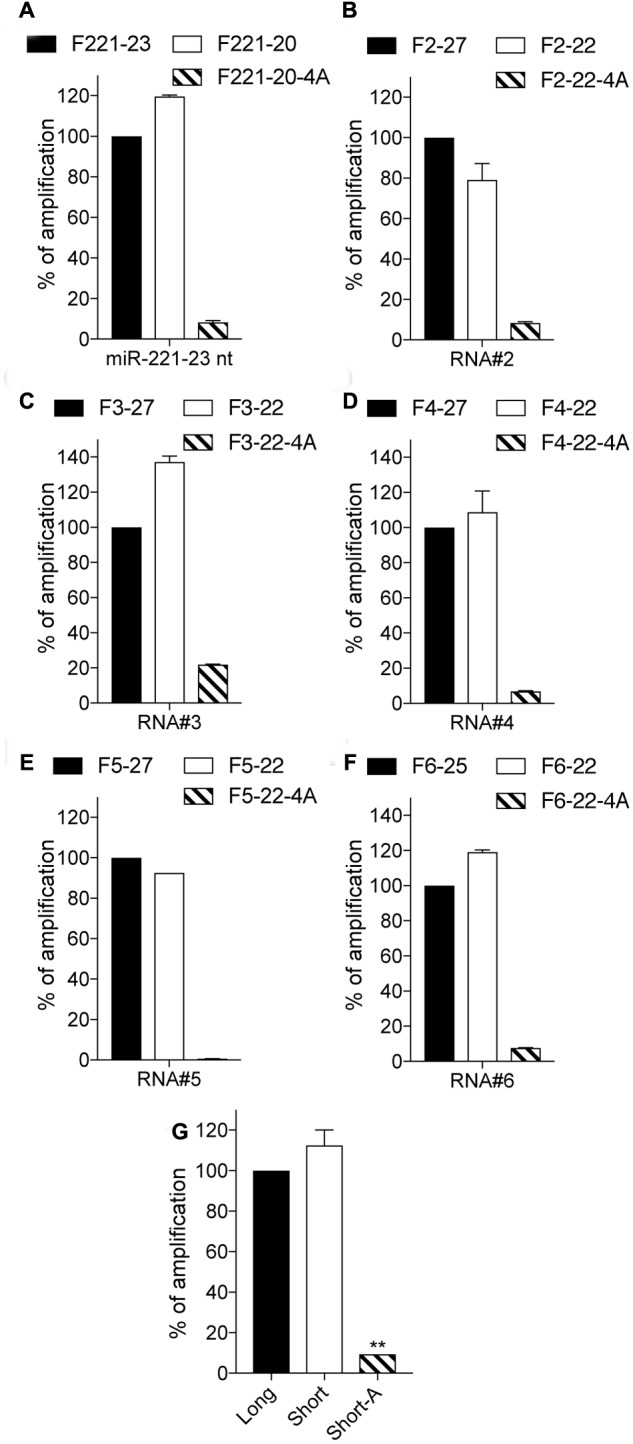
3′-end A-extension decreases off-target amplification of long isoforms. **(A–F)** Indicated RNA sequences were polyadenylated, reverse transcribed, and measured by RT-qPCR. For each RNA, the amplification values are normalized to the Cq value obtained for the non-modified 23, 25 or 27 nt primer, and are displayed as percentages (mean ± SEM is shown). The forward primer names start with an “F”, followed by the RNA number and are ended by the length of the isomiR there are designed to amplify, with “4A” denoting a 4A 3′-terminal stretch. **(A–F)** Data shown is averaged from two independent RT-qPCR analyses. **(G)** Average amplification for the 6 RNAs is calculated (mean ± SEM and unpaired Mann–Whitney *U* test compared to the long forward primer amplification is shown). ^∗∗^*P* ≤ 0.01.

To broaden our observations, we next assessed the capacity of 4A-polyadenylation RT-qPCR to decrease amplification of longer RNAs, relying on miR-221-3p 23 nt and a set of unrelated 5 additional 25 or 27 nt long RNA sequences (**Figures 3A–F** and **Table 2**). In all cases, amplification of longer sequences by the short forward primer was at least nearly as efficient as with that of the long primer. However, 4A-modified short forward primers had a significantly decreased capacity to amplify the longer sequences, averaging a 90% decrease across these 6 RNAs (**Figure 3G**).

### 4A-Modification and Sequence-Specific Amplification

**FIGURE 4:**
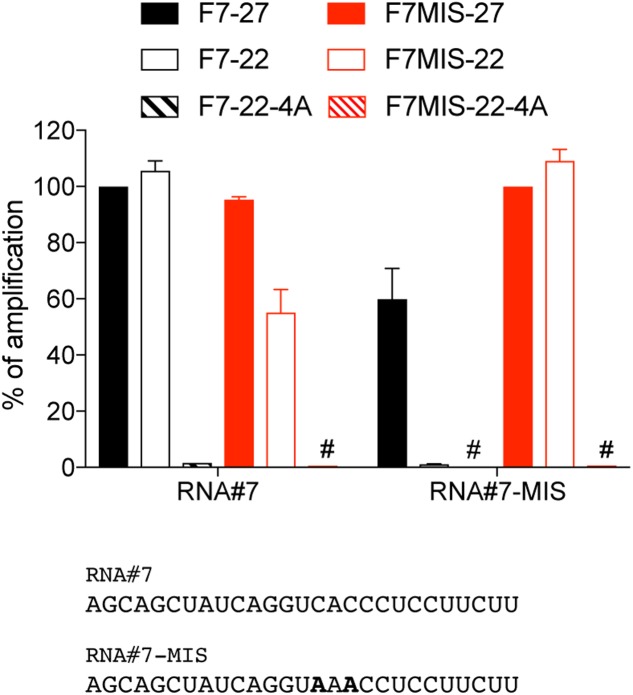
4A-modification and sequence-specific amplification. RNA#7 and RNA#7-MIS differ by two central nucleotides, shown in bold. The amplification values are normalized per RNA, to the Cq value obtained for the 27 nt primer targeting RNA#7 (F7-27) or RNA#7-MIS (F7MIS-27), and are displayed as percentages. The forward primer names start with an “F”, followed by the RNA name and are ended by the length of the isomiR there are designed to amplify, with “4A” denoting a 4A 3′-terminal stretch. Data shown is averaged from two independent RT-qPCR analyses (mean ± SEM is shown). #: denotes amplification values smaller that 1%.

Directly owing to the miRNA sequence they match, the design of forward miRNA primers used in polyadenylation-based RT-qPCRs limits their specificity of amplification. While using backbone modifications such as LNA can help circumvent this, we wanted to assess here how designing shorter forward primers would impact on their specificity toward closely related sequences. For this purpose we compared the amplification of two 27 nt RNA sequences with central 2 nt mismatches (“CAC” > “AAA”), by 22 nt primers with and without the 4A-modification (**Figure 4**). While the longer forward primers amplified both related sequences with little discrimination, shortening the primer to 22 nt enhanced the specificity to the target RNA, probably due to a lower Tm and a greater impact of mismatches on duplex formation. The impact of shortening was most pronounced for the amplification of the “AAA” mutant, in line with this concept (**Figure 4**, see RNA#7-MIS amplification with F7-22). Critically, the 4A-modification potentiated even further the selectivity of the 22 nt primers (as seen with F7MIS-22-A amplification of RNA#7, compared to F7MIS-22), indicating that it did not compromise specificity of amplification.

### Validation of 4A-Modification in Biological Samples

**FIGURE 5:**
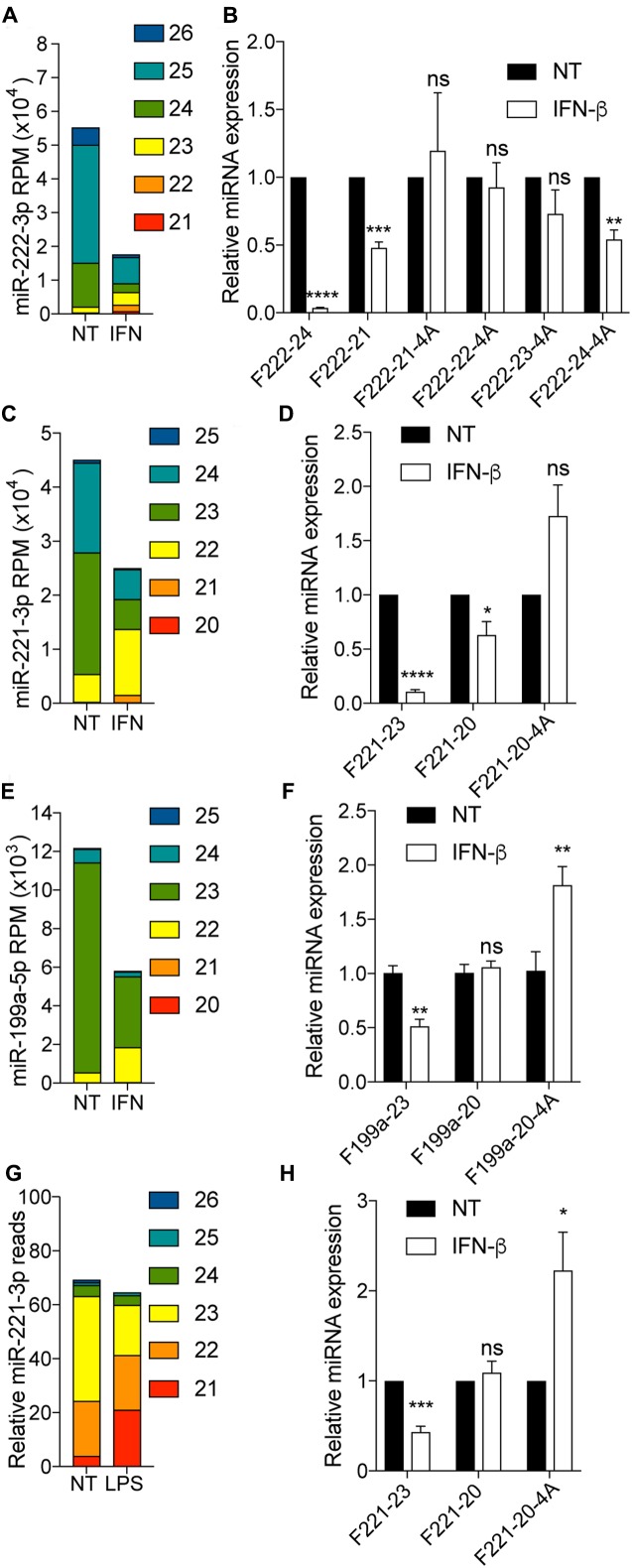
4A-modification helps distinguish isoform-specific responses in biological samples. **(A–F)** Human fibroblasts were treated with 1000 IU/ml of recombinant human IFN-β for 24 h prior to total RNA purification. **(A,C,E)** Small RNA-Seq analysis was performed in biological triplicate, and read count per million (RPM) calculated for each isoform of miR-222-3p **(A)**, miR-221-3p **(C)** and miR-199a-5p **(E)**, based on their length from the canonical 5′-end as per Nejad et al. (2018). The data shown is averaged from biological triplicate. **(B,D,F)** isomiR levels measured with indicated forward primer were reported to U6 RNA. The forward primer names start with an “F”, followed by the miRNA number, and are ended by the length of the isomiR there are designed to amplify, with “4A” denoting a 4A 3′-terminal stretch. Data is shown relative to NT for each isoform-RT-qPCR. Data is averaged from three independent experiments (±SEM and unpaired *t*-tests are shown relative to NT condition for each isomiR/detection method). **(G,H)** BMDMs were stimulated with 100 ng/ml LPS **(G)** or 1000 IU/ml of recombinant mouse IFN-β **(H)** for 24 h. **(G)** Targeted small RNA sequencing of miR-221-3p isoforms and snoRNA 202 was carried out in one sample for each condition, and the number of reads for miR-221-3p reported to 100 reads mapped to snoRNA-202. **(H)** IsomiR levels of miR-221-3p measured with indicated forward primer were reported to U6 RNA. Data is shown relative to NT for each isoform-RT-qPCR. Data is averaged from three independent experiments (±SEM and unpaired *t*-tests are shown relative to NT condition for each isomiR/detection method). ^∗^*P* ≤ 0.05, ^∗∗^*P* ≤ 0.01, ^∗∗∗^*P* ≤ 0.001, ^∗∗∗∗^*P* ≤ 0.0001, ns, not significant.

Relying on small RNA-Seq analyses, we have recently discovered that stimulation of human fibroblasts by interferon (IFN)-β promoted a change in the stoichiometry of miR-221-3p, miR-222-3p and miR-199a-5p isomiRs, leading to decreased levels of isoforms greater than 23/24 nt, while 20-22 nt isoforms where rather induced, with an overall decrease of miR-221-3p miR-222-3p and miR-199a-5p total abundance (Nejad et al., 2018) (**Figure 5**). Critically in these samples, the abundance of the 20–22 isoforms was about one order of magnitude lower than that of >22 nt isoforms (Nejad et al., 2018) (**Figures 5A,C,E**). As such, off-target amplification of the more abundant >22 nt isoforms of these miRNAs by polyadenylation RT-qPCR would be expected to mask changes specific to the 20–22 nt isoforms upon IFN-β stimulation in human fibroblasts. Relying on total RNA from IFN-β stimulated fibroblast, we first compared the amplification of 4A-modifed primers targeting miR-222-3p 21 to 24 nt, to that of unmodified primers. In line with synthetic RNA amplification, the unmodified F222-24 primer revealed a strong decrease of the isoforms amplified upon IFN-β stimulation, matching the strong decrease observed for miR-222-3p isoforms >23 nt (**Figures 5A,B**). Conversely, the unmodified F222-21 primer failed to reflect the increase of miR-222-3p 21/22 nt observed, and rather reflected the global decrease seen across the more abundant isoforms (**Figures 5A,B**). The 4A-modifed 21-23 nt primers increased specificity toward the shorter isoforms of miR-222-3p which were not significantly decreased by IFN-β, while the 4A-modifed 24 nt primer displayed a significant decrease of it targets. We note that F222-23-4A amplification was rather reduced by IFN-β, in line with the fact that this primer also amplifies the very abundant 24-25 miR-222-3p isoforms (**Figure 2D**), which are greatly reduced upon stimulation (**Figures 5A,B**). Importantly in these samples, amplification with F222-24 was more efficient at detecting the IFN-β-driven decrease than its 4A-modified counterpart, probably owing to the enhanced off-target amplification of isoforms shorter than 24 nt by F222-24-4A (as suggested in **Figure 2**). 4A-modifed 20 nt primers increased specificity toward the shorter isoforms of miR-221-3p and miR-199a-5p which were not significantly decreased by IFN-β (**Figures 5D,F**), therefore aligning with our RNA-Seq studies (**Figures 5C,E**). Critically, we have also demonstrated that the effect of IFN-β was not limited to human fibroblasts and could be recapitulated in mouse bone marrow derived macrophages (BMDMs) treated with LPS or IFN-β (Nejad et al., 2018) (LPS driving production of IFN-β in this system). As such, miR-221-3p targeted RNA-Seq of BMDMs treated with LPS mirrored the observations from the human fibroblasts (miR-221-3p 21 nt was increased while miR-221-3p 23 nt was decreased) (**Figure 5G**). The 4A-modified 20 nt miR-221-3p primer demonstrated a significant increase of expression upon IFN-β treatment, otherwise not detected with the unmodified F221-20 primer (which rather reflected the overall global miR-221-3p concentration, mostly unchanged by the treatment) (**Figures 5G,H**). These results collectively suggest that the 4A-modification can be used to distinguish changes in long and short isoforms levels due to stimulation, by polyadenylation RT-qPCR.

## Discussion

Over the past decade, many approaches have been developed to detect miRNAs by RT-qPCR (Mestdagh et al., 2008, 2014; Benes et al., 2015; Niu et al., 2015; Jin et al., 2016; Androvic et al., 2017; Shigematsu et al., 2018). While differing slightly between commercial suppliers, the two predominant approaches are the stem-loop based (e.g., Thermofisher’s Taqman^TM^ miRNA assays and Bioline’s EPIK^TM^ miRNA Select Assays) and the polyadenylation RT-qPCR (e.g., Exiqon/Qiagen miRCURY^TM^ LNA^TM^miRNA PCR System, Quantabio’s qScript, Clontech’s miR-X, and Thermofisher’s Taqman Advanced miRNA assays). While polyadenylation-based approaches allow the user to measure many different miRNAs from the same reverse transcription reaction (with a universal reverse primer), stem-loop strategies are usually restricted to the measure of a few target miRNAs, with specific stem-loops used during RT for each miRNA, although these can be also be pooled (Le Carre et al., 2014).

In this work, we investigated the capacity of polyadenylation RT-qPCR relying on DNA primers to distinguish between 3′-end isoforms of a same miRNA family. Our analysis of synthetic miR-222-3p isoforms varying between 21 and 25 nt demonstrated that forward primers targeted toward shorter isoforms could also amplify longer ones, underlining that short forward primers have the advantage of amplifying the full spectrum of a family’s 3′-end isoforms. Critically, Taqman stem-loop and miRCURY LNA miRNA assays also displayed a lack of specificity toward the 21 nt isoform of miR-222-3p. This was surprising for the latter technology, also based on polyadenylation RT-qPCR, given that the reverse primer used encompasses the junction between the polyadenylated tail and the 3′-end of the miRNA targeted.

In addition, primers targeted to long isomiRs failed to detect isomiRs lacking 2 or more 3′-end nucleotides. A similar observation has been made by us and others with Taqman stem-loop RT-qPCR and linker-adapter RT-qPCR (Androvic et al., 2017; Nejad et al., 2018). We propose that this specificity of longer primers is directly related to the poor annealing of the 3′-end region of the primer to shorter isomiRs, hindering 5′-3′ polymerase activity (Simsek and Adnan, 2000). Although such effect can be reduced with different RT-qPCR approaches (Androvic et al., 2017), it may lead to unintended isomiR bias with Taqman stem-loop RT-qPCR and polyadenylation-based RT-qPCR, by hampering the detection of >1 nt shorter isoforms (Nejad et al., 2018). With 20% of all human miRNAs in miRBase V21 defined as >22 nt, this may impact interpretation on a significant proportion of miRNA families, for which the 20–21 nt isoform can be predominant in specific cell types (as seen with miR-221-3p, miR-222-3p, miR-125a-5p, and miR-107, to cite a few) (Juzenas et al., 2017).

Critically, we establish that addition of 2 or more adenosine residues at the 3′-end of the forward primer targeted to short miRNA isoforms (20–21 nt), significantly decreases amplification of >2 nt longer variants (as seen with miR-221-3p and miR-222-3p). This 4A-modification approach was much more specific toward shorter isoforms than Taqman stem-loop and miRCURY LNA miRNA assays, in addition to being very inexpensive. Combined with primers targeted to isoforms >22 nt, this 4A-modification allowed us to validate selective changes in isoform profiles previously measured by RNA-Seq, in human and mouse cells (Nejad et al., 2018). The 4A-modification facilitated detection of less abundant short isoforms, which change of expression would otherwise be masked by more abundant longer isoforms. Nonetheless, although clearly useful to identify changes between long and short 3′-end isoforms (with ≥ 3 nt difference), this approach may not be specific enough to reveal changes limited to single isoforms (albeit the abundance of the isoform would also be at play). In addition, this strategy cannot be used to distinguish 5′-end isomiRs. Alteration of the forward primer 3′-end used in amplification may, however, also be used in the context of a 5′-end linker ligated to the isomiRs (as seen in the TaqMan Advanced miRNA cDNA Synthesis Kit), to provide such 5′-end isomiR selectivity.

This strategy can readily be applied with commercial polyadenylation RT-qPCR kits relying on user-designed forward primers, such as the miR-X or the qScript kits, and custom enzymatic mixes relying on polyadenylase tailing (Niu et al., 2015). It may also be implemented in other polyadenylation RT kits where custom design of the forward primers is possible. We note, however, that the 4A-modification presented here decreased the selectivity of primers targeted toward longer RNAs (increasing off-target amplification of shorter ones), and should therefore be restricted to detect shorter isoforms (19-21 nt), while primers targeted to longer isoforms (23–25 nt) should preferentially be non-modified (compare **Figures 1A**, **2D**). This effect of the 4A-modification on longer primers possibly relates to the binding of the 4A-stretch to the poly-A tail of shorter isoforms, restoring enough 3′-end stability for the polymerase to start replication. Conversely, when users wish to measure all the isoforms of a miRNA family at once, independent of their length, they should rely on forward primers targeted to 19–20 nt isoforms, rather than the current miRBase definition of miRNA canonical length – which is clearly not reflective of every tissue/cell line (Siddle et al., 2015; Juzenas et al., 2017; Yu et al., 2017). Nonetheless, some miRNAs may be more amenable to this approach than others, as we observed that miR-199a-5p amplification with a non-A modified 20 nt forward primer did not reflect the global decrease of this miRNA family after IFN-β treatment.

## Conclusion

We show that the method described here helps confer selectivity of RT-qPCR detection to isomiRs of varying 3′-end length. We demonstrate its capacity to distinguish between two 3′-end isomiR species, as long as these differ in length by 3 or more nucleotides. Our studies and those of others indicate that such length variation can be induced by cell-stimulation, and bacterial infections (Siddle et al., 2015; Nejad et al., 2018). In addition, recent evidence suggests that there are key functional differences between 24 and 25 nt isoforms compared to the 21 nt isoform of miR-222-3p, possibly relating to the fact that isoforms >23 nt are predominant nuclear localized (Yu et al., 2017). While not specific enough to distinguish each isomiR of a miRNA family, which may be achieved through new techniques (Shigematsu et al., 2018), our approach provides an easy and cost-efficient strategy to define whether long and short variants of a same miRNA family similarly respond to stimulation or disease context. It may also be readily implemented in miRNA-profiling studies, complementing small RNA-Seq approaches in the identification of novel disease biomarkers (Telonis et al., 2017).

## Author Contributions

CN helped design, performed, and analyzed all the experiments, and helped write the manuscript. GP helped with experimental design, performed cell culture studies, and helped write the manuscript. MB helped with the design and synthesis of all synthetic RNAs. MG conceived and coordinated the study, designed and analyzed the experiments, and wrote the manuscript. All authors reviewed the results and approved the final version of the manuscript.

## Conflict of Interest Statement

MB is employed by Integrated DNA Technologies, Inc., (IDT) which offers reagents for sale similar to some of the compounds described in the manuscript. IDT is, however, not a publicly traded company and the author does not personally own any shares/equity in IDT. The other authors declare that the research was conducted in the absence of any commercial or financial relationships that could be construed as a potential conflict of interest.
